# YBX1 Promotes Drug Resistance in Hepatocellular Carcinoma and Serves as a Potential Therapeutic Target

**DOI:** 10.21203/rs.3.rs-9316539/v1

**Published:** 2026-04-07

**Authors:** Manish Tripathi, VEERABABU NAGATI, Dennis Kwabiah, Yamile Abuchard Anaya, Ana Pazzi, Salique Shaham, Mohammad Hussain, Ricardo Bracho, Kyle Doxtater, Samantha Lopez, Sophia Leslie

**Affiliations:** The University of Texas Rio Grande Valley; The University of Texas Rio Grande Valley; The University of Texas Rio Grande Valley; The University of Texas Rio Grande Valley; The University of Texas Rio Grande Valley; The University of Texas Rio Grande Valley; The University of Texas Rio Grande Valley; The University of Texas Rio Grande Valley; The University of Texas Rio Grande Valley; The University of Texas Rio Grande Valley; The University of Texas Rio Grande Valley

## Abstract

Drug resistance has emerged as a significant factor contributing to the dismal prognosis of patients with hepatocellular carcinoma (HCC). Since tyrosine kinase Inhibitors (TKIs) are the standard first-line therapy for advanced HCC, however, its effectiveness is significantly hindered by the development of drug resistance, the mechanisms of which are still not fully understood. This study aims to investigate the potential role of the transcription factor YBX1 in mediating drug resistance and to validate it as a potential therapeutic target in HCC. We identified increased YBX1 levels in human HCC patient cohorts and found that it is associated with tumor aggressiveness, metastasis, and poor survival, and is a key transcription factor contributing to drug resistance. Our results show that YBX1 overexpression confers sorafenib resistance in HCC. Elevating YBX1 levels in HCC cell lines increased cell survival, viability, and sorafenib IC50 values, as well as tumorigenic features and drug resistance markers. Conversely, siRNA-mediated knockdown of YBX1 reduced these effects. Sorafenib-resistant cells exhibited increased YBX1 and resistance markers. Inhibiting YBX1 significantly decreased the viability of resistant cells. In vivo studies demonstrated that inhibiting YBX1 with the small-molecule SU056 reduces tumor size. Thus, YBX1 is a promising target for extending drug resistance in HCC.

## INTRODUCTION

HCC is a predominant form of primary liver cancer globally, with over half a million new cases diagnosed every year [1–3]. According to recent global statistics, there were 906,000 incidences of HCC globally and 830,000 fatalities attributed to this disease, making it the sixth most commonly diagnosed form of cancer and the third primary cause of cancer-related mortality [4]. Between 2020 and 2040, the incidence and death rate of HCC are expected to rise by 55% and 56.4%, respectively [5]. The mortality rate of HCC is experiencing a more rapid increase in the United States [1]. The United States is projected to have 42,240 new cases of liver cancer in 2025, with an anticipated fatality rate of 30,090 [6]. This corresponds to an approximate death rate of 70% related to HCC. The Surveillance, Epidemiology, and End Results (SEER) database links HCC to chronic hepatitis B virus and hepatitis C infection [1]. Although there have been improvements in the treatment methods for HBV and HCV in the United States, incidences of HCC and its related death rate continue to be widespread in the country [1, 7]. Early detection of HCC enables patients to benefit from potentially curative interventions [2, 8, 9]. Liver transplantation and hepatectomy are treatment options that can cure HCC when diagnosed early [8, 9]. However, in its late stages, the disease is difficult to treat due to a lack of effective treatment options [2, 10, 11].

The emergence of drug resistance in cancer poses a significant challenge despite notable advances in treatment over the past few decades [12]. Resistance to new targeted therapies remains a critical issue, contributing to approximately 90% of cancer-related deaths [12]. Drug resistance and treatment ineffectiveness arise from various factors, including genetic mutations, epigenetic alterations, and other cellular and molecular mechanisms [12]. Although recent advances in novel chemotherapy have shown remarkable efficacy in inhibiting cancer cell proliferation and progression, most patients eventually develop resistance as treatment progresses. To improve therapeutic outcomes and facilitate the development of novel, effective therapeutics, the mechanisms underlying drug resistance need to be better elucidated [12].

Sorafenib is a multikinase inhibitor well known for its efficacy in suppressing tumor growth and angiogenesis and promoting apoptosis across a wide spectrum of cancers, including breast, colorectal, and advanced renal cell carcinomas, as well as differentiated thyroid cancers. [11]. It exerts its antitumor effects on multiple solid cancers by blocking kinase pathways [10]. It targets serine-threonine kinases Raf-1 and B-Raf, as well as the receptor tyrosine kinase activity of vascular endothelial growth factor receptors (VEGFRs) 1, 2, and 3, and platelet-derived growth factor receptor β (PDGFR-β) [10, 13]. Preclinical data convincingly demonstrate sorafenib’s strong antiproliferative activity against liver cancer cell lines, as evidenced by its inhibition of tumor angiogenesis and key signaling pathways and by the concurrent induction of apoptosis in a human hepatocellular carcinoma xenograft mouse model. [10]. Sorafenib is approved as an effective first-line treatment for late-stage hepatocellular carcinoma (HCC). However, approximately 30% of patients respond to sorafenib, with the majority developing resistance within 6 months of treatment [14]. Sorafenib shows limited long-term efficacy in HCC due to frequent resistance [3, 15]. As a result, despite its use in treating HCC, the disease remains prone to relapse, leading to a discouraging prognosis [2, 10, 16].

YBX1 is a key transcription factor and a prominent oncoprotein involved in various cancers [17, 18]. As a member of the cold-shock protein superfamily, YBX1 binds nucleic acids, interacting with both DNA and RNA [19–22]. It is involved in transcriptional activation, DNA repair, replication, and several other cellular processes [18, 20]. YBX1 significantly promotes cancer cell proliferation [23, 24]. Within the nucleus, it functions as a transcription factor, while in the cytoplasm, it acts as an RNA-binding factor [20]. YBX1 controls transcription via the Y-box binding site (inverted CCAAT box) and additionally regulates mRNA stability and translation within the cytoplasm [25]. YBX1 is recognized as a prognostic clinical biomarker and is associated with unfavorable clinical outcomes across various cancers, including breast, lung, bladder, prostate, and diffuse large B-cell lymphoma [19, 20, 26]. YBX1 predominantly resides in the cytoplasm but translocates to the nucleus under stress [27]. The nuclear localization of YBX1 correlates with the aggressiveness and multidrug resistance of cancer cells, underscoring the need for insight into the regulatory mechanisms governing its subcellular distribution [27]. It is identified as a key regulator of drug resistance in breast and colorectal cancers [2, 17, 26]. Additionally, YBX1 confers resistance to gefitinib, gemcitabine, and cisplatin in lung adenocarcinoma, bladder cancer, and neuroblastoma, respectively [26]. An extensive study of YBX1 has established its role in drug resistance across multiple cancer types. However, its role in sorafenib resistance in hepatocellular carcinoma (HCC), one of the most prevalent cancer types globally, remains underexplored [2]. This study aims to investigate the role of YBX1 and its potential association with sorafenib resistance in HCC.

## MATERIALS AND METHODS

### Data collection

Publicly available datasets were used for this study. Gene expression data for YBX1 in liver hepatocellular carcinoma (LIHC) were retrieved from The Cancer Genome Atlas (TCGA) via the UALCAN Cancer Data Analysis Portal (https://ualcan.path.uab.edu/). Analysis of YBX1 mRNA expression, progression, metastasis, and survival was performed in the HCC cohort using the TCGA database via https://ualcan.path.uab.edu/ [28]. Protein expression data for YBX1 in HCC tissues versus normal liver samples were obtained from the Clinical Proteomic Tumor Analysis Consortium (CPTAC), also accessed through the UALCAN portal (https://ualcan.path.uab.edu/cgi-bin/ualcan-res-prot.pl). These datasets were used to assess differential expression patterns at both the transcriptomic and proteomic levels in liver cancer [28, 29].

### Cell culture

HCC cell lines were obtained from the American Type Culture Collection (ATCC). Cells were maintained in Eagle’s Minimum Essential Medium (EMEM) (ATCC), supplemented with 10% fetal bovine serum (FBS) (MidSci) and 1% penicillin-streptomycin (Thermo Fisher Scientific). Cultures were incubated at 37°C in a humidified atmosphere containing 5% CO_2_.

### Generation of Stable GFP-Tagged YBX1 Overexpression and Knockdown Cell Lines

GFP-tagged YBX1, cloned into the lentiviral vector pLenti-GIII-CMV-GFP-2A-Puro (Abmgoods; Figure S2A), driven by a CMV promoter, was used for the overexpression in a cell line. For knockdown experiments, pLenti-siRNA-GFP (Abmgoods; Figure S2B) was used, containing one of four different siRNA sequences targeting human YBX1 (listed in Supplementary Table S3), along with a scrambled siRNA control. The siRNA cassettes were driven by either U6 or H1 promoters, and both vectors encoded GFP and puromycin resistance genes for selection and enrichment. Forty-eight hours post-transfection, cells were subjected to puromycin selection (1–2 μg/mL) for 3–4 days to select stable cells. Following drug selection, fluorescence-activated cell sorting (FACS) was performed using a SONY cell sorter to enrich GFP-positive cells. As shown in *Figure S3A*, YBX1-overexpressing cells exhibited approximately 8.1% GFP positivity after puromycin selection. In knockdown lines (*Figure S3B*), approximately 1.2% of cells were GFP-positive. To further improve knockdown efficiency, the top 20% of GFP-expressing cells (representing 0.81% of total events) were sorted and expanded to establish stable knockdown populations. All sorted cell populations were continuously maintained under puromycin selection and subsequently expanded for downstream validation and experimental use.

### Cell viability assay

Cell viability was assessed using the 3-(4,5-dimethylthiazol-2-yl)-2,5-diphenyltetrazolium bromide) MTT assay. A total of 10,000 cells per well were seeded into 96-well plates (VWR, Cat# 10861-562). After 24 hrs., cells were treated with varying concentrations of the test compound, dissolved in DMSO, with the final DMSO concentration maintained at ≤ 0.1% to avoid solvent-induced cytotoxicity. Treatments were applied at designated time points depending on the experimental design. Following treatment, 20 μL of MTT solution (5 mg/mL in 1× PBS) was added to each well, and the plates were incubated at 37°C in a 5% CO_2_ incubator for 3 hours. After incubation, the media and MTT solution were removed, and the resulting formazan crystals were solubilized in 100 μL of DMSO per well. Absorbance was measured at 570 nm using a Varioskan Lux multimode plate reader (Thermo Fisher Scientific). Raw absorbance values were normalized to untreated controls and expressed as percent cell viability. Each condition was tested in technical quadruplicate, and all experiments were independently repeated at least three times.

### Tissue Microarray and Immunohistochemistry

A human liver hepatocellular tissue microarray containing 48 pairs of HCC and matched adjacent normal tissue microarray slides (LV8011a) was purchased from US Biomax, Inc., MD, USA. The demographic and pathological characteristics are presented in Table 1. The immunohistochemistry assay was performed as previously described [30].

### Development of drug (sorafenib) resistant cells

Sorafenib-resistant hepatocellular carcinoma cell lines were generated through stepwise drug adaptation. Baseline IC_50_ values for each parental cell line were first established using dose-response assays (see Supplementary Figure S4). To induce resistance, cells were initially treated with low concentrations of sorafenib for 72 hours, followed by a recovery phase in drug-free Eagle’s Minimum Essential Medium (EMEM) until they reached approximately 70% confluency. This cycle of sorafenib exposure and recovery was repeated, with gradually increasing drug concentrations, allowing for selection of resistant subpopulations. This incremental selection continued until the cells exhibited sustained proliferation and survival in the presence of 10 μM sorafenib, a concentration exceeding the initial IC_50_. Once resistance was established, cells were maintained in EMEM supplemented with 10 μM sorafenib to preserve the resistant phenotype. These cell populations were designated as sorafenib-resistant (SRC) lines for downstream experiments.

### Invasion and migration assay

Transwell assays were performed to evaluate migration and invasion. Cells were harvested, washed with phosphate-buffered saline (PBS), and resuspended in serum-free Eagle’s Minimum Essential Medium (EMEM) at a concentration of 1 × 10^5^ cells/mL. For each assay, 100 μL of the cell suspension was added to the upper chamber of a Transwell insert (8 μm pore size), while the lower chamber was filled with 600 μL of EMEM supplemented with 30% fetal bovine serum (FBS) to serve as a chemoattractant. Following a 24-hour incubation at 37°C in a humidified 5% CO_2_ incubator, non-migrated cells on the upper surface of the membrane were gently removed using a cotton swab. The membranes were then fixed with 4% paraformaldehyde for 30 minutes and stained with 0.5% crystal violet for 5 minutes at room temperature. Membranes were washed with PBS, and images of migrated cells on the lower surface were captured using a light microscope at 20× magnification from five randomly selected fields per insert. For invasion assays, the protocol was identical, except that Transwell inserts were pre-coated with Matrigel (Corning) and incubated at 37°C for 2 hours prior to cell seeding to simulate the extracellular matrix barrier.

### Cell proliferation assay

Cell proliferation was measured using the MTT assay. Cells were seeded into 96-well plates at a density of 10,000 cells per well in complete growth medium and incubated at 37°C in a humidified incubator with 5% CO_2_. At 24 and 48 hours post-seeding, 10 μL of MTT reagent (5 mg/mL in PBS) was added to each well, followed by a 2-hour incubation to allow metabolically active cells to form MTT formazan crystals. After incubation, absorbance was recorded at 570 nm using a microplate reader (Varioskan Lux, Thermo Fisher Scientific). Background absorbance from media-only wells was subtracted, and values were normalized to the 24-hour control group to assess relative proliferation. Results were expressed as a percentage of viable cells relative to baseline. Each condition was tested in technical quadruplets, and the experiment was repeated at least 3 times in independent biological replicates to ensure reproducibility.

### Colony formation assay

A total of 1,000 cells per well were seeded into 6-well plates and cultured under standard conditions (37°C, 5% CO_2_) for 14 days to allow colony development. Culture medium was replenished every 3–4 days. At the end of the incubation period, colonies were fixed with 4% paraformaldehyde for 30 minutes at room temperature, then stained with 0.1% crystal violet for 15 minutes. Excess stain was removed by gentle rinsing with distilled water, and plates were air-dried. Stained colonies were counted manually under a light microscope to determine the total number of colonies formed per well. Each condition was tested in triplicate and repeated at least 3 times in independent experiments.

### Western blot

Total protein was extracted from cultured cells using RIPA buffer containing protease and phosphatase inhibitors. Protein concentrations were quantified using the Bradford assay, and equal amounts of protein were resolved by SDS-PAGE. Proteins were then transferred onto 0.2 μm PVDF membranes (Millipore) using a semi-dry system (Idea Scientific). Membranes were blocked with blocking buffer (5% non-fat milk in TBST) for 1 hour at room temperature, then incubated with the appropriate primary antibodies overnight at 4°C. The following primary antibodies were used: YBX1, MDR1, PD-L1, and CD44 (see Supplementary Table S1). The next day, membranes were washed and incubated with species-specific HRP-conjugated secondary antibodies for 1 hour at room temperature. Protein bands were visualized using enhanced chemiluminescence (ECL) and imaged with a digital imaging system.

### RNA isolation, cDNA synthesis, and qRT-PCR

Total RNA was extracted using TRIzol^™^ Reagent (Life Technologies, Cat# 15596-026) according to the manufacturer’s protocol. The concentration and purity of RNA were assessed using a nanodrop. A total of 2 μg of RNA was reverse transcribed into complementary DNA (cDNA) using the Thermo Fisher Scientific cDNA Synthesis Kit, following the manufacturer’s instructions. Quantitative real-time PCR (qRT-PCR) was performed using the Bio-Rad CFX96 Real-Time PCR Detection System (C1000 Touch^™^ Thermal Cycler). Primers were designed using the Harvard Primer Bank and synthesized by Integrated DNA Technologies (IDT). Primer sequences used in the study are provided in Supplementary Table 1. Gene expression levels were analyzed using the 2^−ΔΔCt^ method, and results were expressed as fold change relative to control samples. Statistical analysis was performed using a paired t-test with ratio comparison in GraphPad Prism version 10.6, with *p* < 0.05 considered statistically significant.

### Tissue scan cDNA array

A human liver hepatocellular tissue microarray (LVRT101) containing 48 pairs of HCC and matched adjacent normal tissue was purchased from Biomax. Ltd, USA. Demographic and pathological characteristics are given in Table 2. Performed qRT-PCR by using YBX1 primers mentioned in the supplementary table 1.

### Transfection

Cells were seeded into 12-well plates, and transfections were performed the following day using Lipofectamine^®^ LTX with PLUS^™^ Reagent (Invitrogen) according to the manufacturer’s protocol. The expression plasmids used included pLenti-GIII-CMV-YBX1-GFP-2A-Puro (for YBX1 overexpression) and pLenti-siRNA-GFP (for YBX1 knockdown). For optimization, plasmids were mixed with varying concentrations of Lipofectamine LTX and DNA Plus reagent in Opti-MEM^™^ reduced-serum medium (Thermo Fisher Scientific) in 24-well plates. After a 5-minute incubation at room temperature, the transfection mixture was added dropwise to the cells and incubated for 10 minutes, then replaced with complete medium. Transfection efficiency was evaluated 24–48 hours post-transfection by assessing GFP fluorescence using a fluorescence microscope.

### Mice

Male nude mice (NU/J strain 00219; 4 weeks old) were purchased from the Jackson Laboratory, USA. The mice were housed in a case under specific pathogen-free conditions (ambient temperature of approximately 20–26°C, 40–60% humidity, and 12/12 h dark/light cycles) during the study.

### HCC xenograft models

We conducted *in vivo* experiments to investigate the antitumor effects of sorafenib and the small-molecule YBX1 inhibitor, SU056, in HCC. Previous studies demonstrated that sorafenib at 20mg/kg and SU056 at 10mg/kg inhibit the YBX1 and reduce the tumor growth in triple-negative breast cancer and ovarian cancer [31, 32]. Based on this literature, we ultimately chose a dose of 10mg/kg/d in our pilot experiments to confirm the synergistic effect of sorafenib and SU056 in inhibiting YBX1 and reducing tumor growth. Cultured Huh7 luc cells (2x10^6^) were injected into the flank of BALB/c nude mice. When the tumor diameter reached 5 mm, mice were randomly assigned to 4 groups (4 mice/group) and received the following treatments. Control group, SU056 10mg/kg/d of SU056, 20mg/kg/kg/week sorafenib, and both sorafenib and SU056 combination group. After 3 weeks of treatment, the mice were sacrificed, and the xenograft tumors were removed and weighed.

### Statistical analysis

Statistical analyses were performed using GraphPad Prism software (GraphPad, Inc., LLC, USA version 16.2). The data are presented as indicated in the Figure legend. Statistical significance was calculated using an unpaired two-sided Student’s t-test (for comparison of two groups), or one-way or two-way ANOVA followed by Tukey’s multiple comparisons analysis (for comparison of three or more groups). Data are presented as mean ± SE. The comparison between groups was performed using an unpaired t-test (*p < 0.05; ** p < 0.01; *** p < 0.001; **** p < 0.0001).

## RESULTS

### YBX1 is overexpressed in HCC and correlates with disease progression

**Figure 1:**
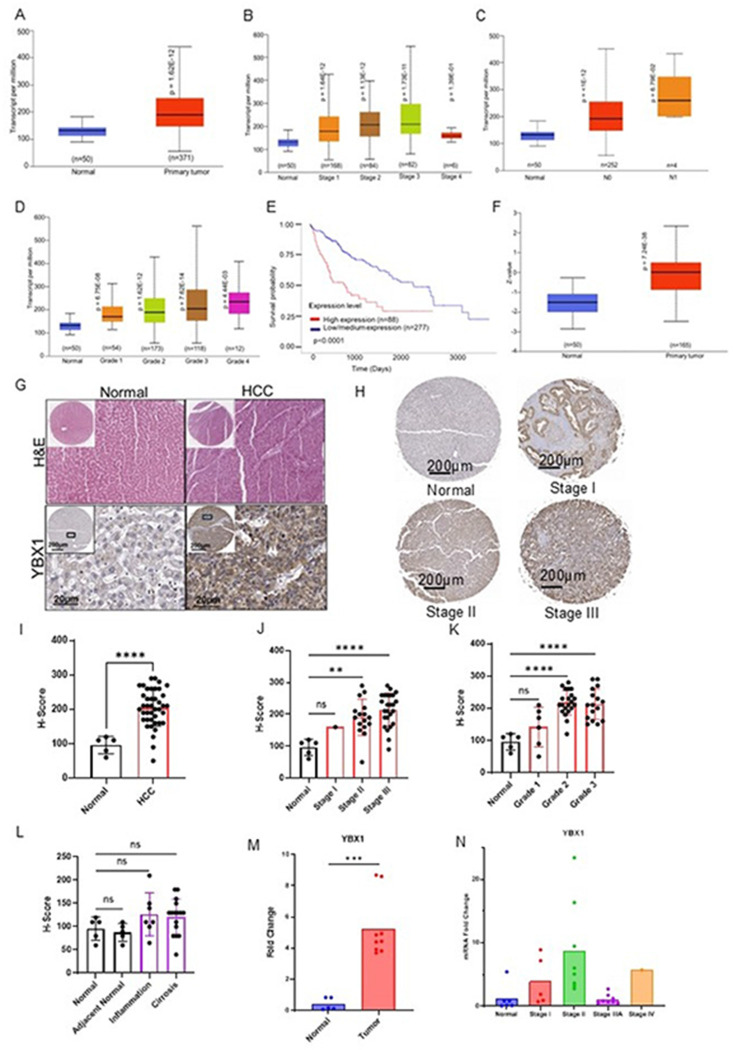
YBX1 is a prognostic factor and is overexpressed in HCC A) mRNA expression of YBX1 is significantly increased in the primary tumor tissues of HCC (n=371) compared to corresponding normal liver samples (n=50), based on TCGA analysis. B) The relationship between YBX1 mRNA levels and tumor stage in HCC patients shows a progressive rise with advancing stages. C) YBX1 mRNA levels categorized by nodal metastasis status: N0 (no regional lymph node metastasis) versus N1 (metastasis in 1–3 regional lymph nodes), compared to normal tissues, in the TCGA analysis. D) YBX1 expression levels across tumor grades (Grade 1–4), indicating increased expression with higher tumor grade, in the TCGA analysis. E) Kaplan-Meier survival analysis reveals significantly poorer overall survival in patients with high YBX1 expression (n=88) versus those with low or medium expression (n=277), in the TCGA cohort (p < 0.0001). F) YBX1 protein levels are markedly upregulated in HCC tumor tissues (n=165) compared to normal liver tissue (n=50), based on TCGA (CPTAC) data. G) Representative histological images from liver tissue microarray (TMA, LV8011a) stained with hematoxylin and eosin (H&E) and YBX1 immunohistochemistry (IHC), showing strong cytoplasmic and nuclear staining in HCC compared to normal liver. H) Immunohistochemical staining of YBX1 in normal liver and HCC tissues stratified by pathological stages (I–III) from a sample cohort of 80. I–L) Quantification of YBX1 IHC staining using H-score (mean ± SD), assessed via one-way ANOVA and Tukey’s post hoc tests: (I) Normal versus HCC tissues; (J) By tumor stage (normal, stage I–III); (K) By tumor grade (normal, grade 1–3); (L) Comparison of YBX1 expression across normal, inflammation, and cirrhosis conditions, showing no significant increase in non-cancerous tissues. M–N) YBX1 mRNA levels analyzed with qRT-PCR in a liver cDNA array comparing normal and tumor tissues, showing: (M) a significant fold increase in tumors; (N) YBX1 mRNA expresssion levels at different stages. Statistical annotations: ns = not significant; *p < 0.05; **p < 0.01; ***p < 0.001; **** p < 0.0001. Scale bars: 20 μm (G), 200 μm (H).

To investigate the clinical relevance of YBX1 in HCC, we analyzed publicly available datasets and patient-derived samples. Analysis of the TCGA dataset revealed that YBX1 mRNA expression was significantly elevated in HCC tissues (n = 371) compared with normal liver tissues (n = 50) (Fig. 1A). Furthermore, YBX1 expression progressively increased from stage I to stage III tumors, with a modest decline in stage IV tumors, suggesting a correlation with tumor progression (Fig. 1B). Elevated YBX1 levels were also significantly associated with nodal metastasis (Fig. 1C), advanced histological grade (Fig. 1D), and worse overall survival (Fig. 1E), suggesting its potential prognostic value. Consistent with transcriptomic data, YBX1 protein expression was significantly higher in primary HCC tumors than in normal tissues in the Clinical Proteomic Tumor Analysis Consortium (CPTAC) dataset (Fig. 1F). To validate these findings, we performed tissue microarray (TMA) analysis using an independent patient cohort (n = 80, LV8011a). Immunohistochemistry revealed strong YBX1 staining in tumor tissues, which was minimal or absent in adjacent normal liver (Fig. 1G–H). The full TMA image is available in Supplementary Figure S1. Quantitative H-score analysis showed a stage-dependent increase in YBX1 protein levels (Fig. 1I), with further stratification revealing significantly higher YBX1 scores in tumors with advanced stage, high grade, inflammation, or cirrhosis (Figs. 1J–L). The patient’s TMA distribution and pathological details are provided in Table 1. Additionally, analysis of a separate HCC cDNA array (LVRT101, n = 48) confirmed that YBX1 mRNA was significantly overexpressed in tumor tissues across various stages (Fig. 1M–N), reinforcing the clinical consistency of YBX1 upregulation in HCC. The demographic and pathological features of the cDNA array cohort (LVRT101) are detailed in Table 2.

### YBX1 promotes sorafenib resistance and drives tumorigenic phenotypes in HCC

Earlier studies have demonstrated that YBX1 overexpression contributes to aggressive malignant traits such as drug resistance. The 50% inhibitory concentration (IC_50_) has been successfully used as a standard indicator of drug resistance in previous studies [33]. We performed an initial profiling of the IC50 values for HCC cell lines treated with sorafenib using the MTT assay (*Figure S4A-D*).

**Figure 2:**
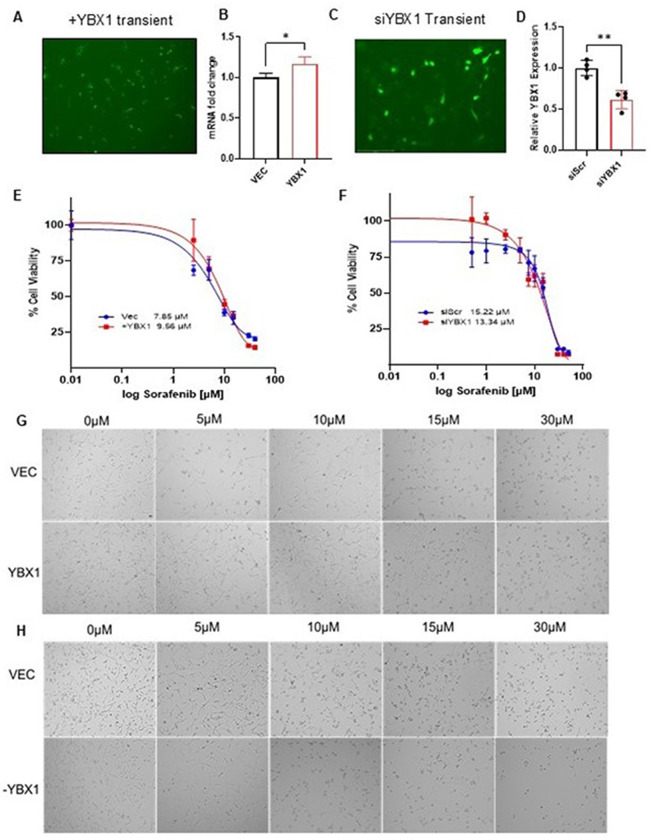
YBX1 mediates sorafenib resistance in HCC cells A–B) SK-Hep1 hepatocellular carcinoma cells were transiently transfected with a GFP-tagged YBX1 plasmid and an empty control vector (VEC). (A) GFP fluorescence confirms successful transfection. (B) qRT-PCR analysis shows significantly increased YBX1 mRNA levels in the YBX1-overexpressing cells compared to the vector control (*p < 0.05). C–D) YBX1 knockdown in SK-Hep1 cells was performed using siRNA plasmid. (C) GFP fluorescence following siYBX1 transfection indicates efficient delivery. (D) qRT-PCR confirms a significant reduction in YBX1 mRNA levels in siYBX1-transfected cells compared to siScramble (**p < 0.01). E) Overexpression of YBX1 increases sorafenib IC50, indicating reduced sensitivity to sorafenib. Dose-response curves show sorafenib sensitivity in SK-Hep1 cells overexpressing YBX1 compared to the vector control. Cell viability was measured after 24 hours of drug treatment. Representative IC50 changes show cell viability (%) versus sorafenib concentration (μM) for transiently transfected cell lines. F) YBX1 knockdown using siRNA enhances sorafenib sensitivity in SK-Hep1 cells, as indicated by a leftward shift in the dose-response curve and decreased IC50. Dose-response curves show sorafenib sensitivity in SK-Hep1 cells with siYBX1 compared to siScr control. Cell viability was measured after 24 hours of drug treatment. Representative IC50 changes show cell viability (%) versus sorafenib concentration (μM) for transiently transfected cell lines. G) Representative phase-contrast images show SK-Hep1 control (VEC) and YBX1-overexpressing cells treated with increasing doses of sorafenib (0, 5, 10, 15, 30 μM) for 24 hours. Overexpression of YBX1 increases cell survival compared to the vector control. H) Representative phase-contrast images of SK-Hep1 cells with control (siScr) or YBX1 knockdown (siYBX1) treated with sorafenib at the indicated concentrations for 24 hours. Knockdown of YBX1 reduces cell viability in a dose-dependent manner compared with the siScramble control. These findings support the role of YBX1 as a mediator of sorafenib resistance in HCC cells, where YBX1 overexpression increases IC50 and knockdown restores sorafenib sensitivity. Data are shown as the mean ± SE. The comparison between the two groups was performed using an unpaired t-test (*p < 0.05; ** p < 0.01; *** p < 0.001; **** p < 0.0001).

To investigate the functional role of YBX1 in sorafenib resistance, GFP-tagged YBX1 was transiently overexpressed in SK-Hep1 cells using lentiviral constructs (Supplementary Figure S2), and GFP expression confirmed transfection efficiency (Fig. 2A). qRT-PCR analysis demonstrated successful upregulation of YBX1 mRNA following transfection (Fig. 2B). Functional drug assays revealed that YBX1-overexpressing cells exhibited a higher IC_50_ for sorafenib compared to vector controls (Fig. 2E), and significantly increased cell viability under sorafenib treatment (Fig. 2G), suggesting enhanced resistance. Conversely, YBX1 knockdown was achieved via siRNA-mediated silencing (Fig. 2C–D). Four different siRNA constructs were tried initially. SK-Hep1 siYBX1 cells displayed markedly reduced viability following sorafenib treatment compared to siScr controls (Fig. 2F–H), supporting a sensitizing effect of YBX1 depletion.

### Stable YBX1 expression drives tumorigenic characteristics and increases sorafenib IC50 in HCC

**Figure 3:**
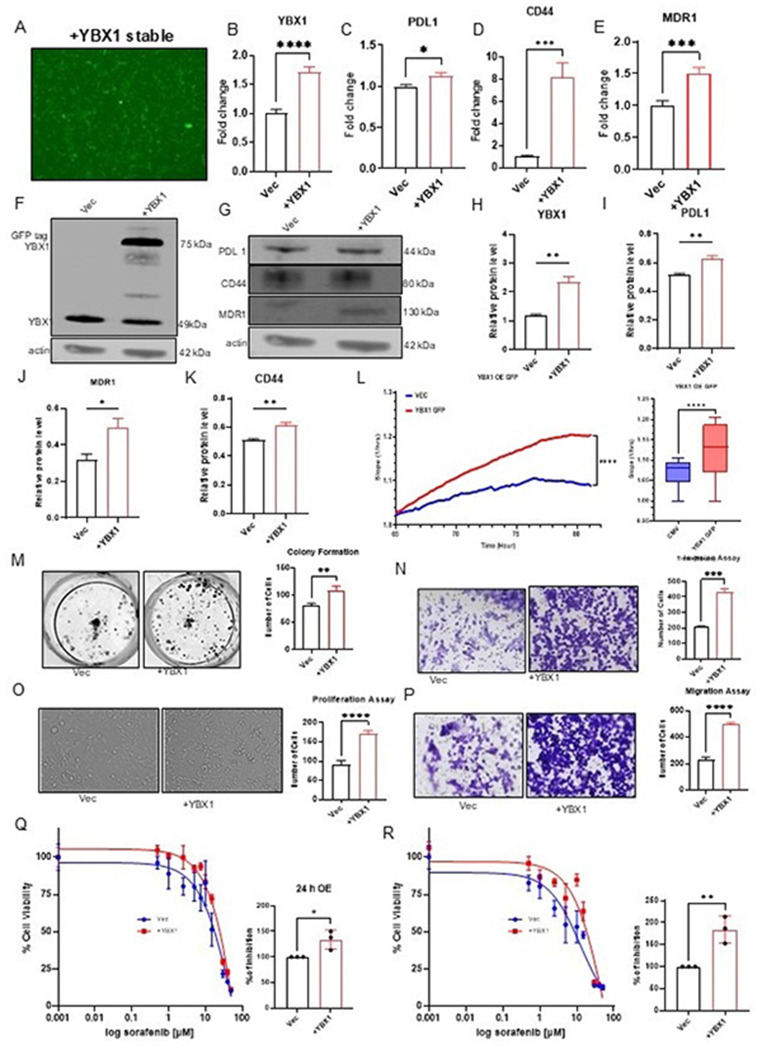
Stable YBX1 expression drives tumorigenic characteristics and increases sorafenib resistance in HCC A–E) YBX1 overexpression and tumorigenic phenotype of SK-Hep1 cells stably overexpressing YBX1 (+YBX1) compared to control vector (Vec). (A) Puromycin-selected, FACS-enriched GFP cells confirm +YBX1 stable cell line. (B–E) mRNA expression levels of YBX1, PD-L1, CD44, and MDR1 are significantly higher in YBX1-overexpressing cells, indicating activation of downstream genes involved in drug resistance and immune evasion pathways. F–K) Western blot analysis of protein expression in SK-Hep1 cells. (F) GFP-tagged YBX1 confirms overexpression. (G) Protein levels of PD-L1, CD44, and MDR1 are elevated in YBX1-overexpressing cells compared to vector controls. (H–K) Densitometric quantification of immunoblots (normalized to β-actin) confirms significant upregulation of YBX1, PD-L1, MDR1, and CD44 in YBX1-overexpressing cells. L) Real-time cell proliferation monitored via xCELLigence assay shows significantly enhanced proliferation (impedance) in YBX1-overexpressing cells versus vector controls. Right panel: box plot summarizing proliferation indices. M) Colony formation assay: representative images and quantification show increased clonogenic survival in YBX1-overexpressing cells. N) Matrigel invasion assay: representative images and quantification display increased invasive capacity in YBX1-overexpressing cells. O) Bright-field images and quantification of the proliferation assay indicate higher cell numbers in YBX1-overexpressing cells. P) Transwell migration assay reveals significantly enhanced migratory ability in YBX1-overexpressing cells compared to vector controls. Q–R) Dose-response curves of sorafenib treatment in SK-Hep1 cells. (Q) 24-hour treatment with sorafenib increases the IC50 in YBX1-overexpressing cells compared to vector-transfected cells. The right panel indicates increased resistance. (R) Similar trends are observed with longer exposure (48 hrs.), confirming that YBX1 overexpression confers sorafenib resistance. Right panels: bar plots showing quantified IC50 shifts. Data are presented as mean ± SE. The comparison between the two groups was performed using an unpaired t-test (*p < 0.05; ** p < 0.01; *** p < 0.001; **** p < 0.0001).

Stable GFP-tagged YBX1-overexpressing lines were further established using puromycin selection and FACS enrichment, as shown in Supplementary Figure S3 and validated by RT-qPCR (Fig. 3A–E). To explore the broader impact of YBX1 on oncogenic properties, YBX1 expression and downstream genes involved in drug resistance [25, 34] were analyzed. Western blotting confirmed a mobility shift in GFP-tagged YBX1, and elevated expression of these resistance markers as compared to vector only control. The overexpression of YBX1 led to upregulation of MDR1, PD-L1, and CD44 at both the mRNA and protein levels (Fig. 3F–K) compared with the vector control. Functionally, YBX1-overexpressing cells exhibited higher colony formation, proliferation, migration, and invasion, as shown by conventional assays and real-time impedance-based XCELLigence analysis (Fig. 3L–P). These results establish YBX1 as a potent driver of both drug resistance genes and aggressive phenotypes in HCC.

To explore whether YBX1 regulates sorafenib sensitivity in HCC, we evaluated the IC_50_ values of YBX1-modulated cell lines using the MTT assay. In YBX1-overexpressing stable cell lines, treatment with increasing concentrations of sorafenib (0.5–50 μM) for 24 and 48 hours showed significantly elevated IC_50_ values compared to empty vector controls. Specifically, at 24 hours, the YBX1-overexpressing cells exhibited an IC_50_ of 24.56 μM, compared with 17.72 μM in controls, representing a 38% increase (Fig. 3Q). At 48 hours, this difference widened, with YBX1-OE cells showing an IC_50_ of 18.47 μM compared to 8.64 μM in controls, which is 113% increase (Fig. 3R). These results indicate that YBX1 overexpression confers a significant increase in sorafenib drug sensitivity at 24 and 48 hrs., implicating it in sorafenib resistance. Collectively, these data indicate that YBX1 is essential for HCC proliferation, progression, and metastasis and plays a critical role in mediating sorafenib drug resistance in HCC.

### Knockdown of YBX1 suppresses oncogenic phenotypes and reduces sorafenib IC50 in HCC

**Figure 4:**
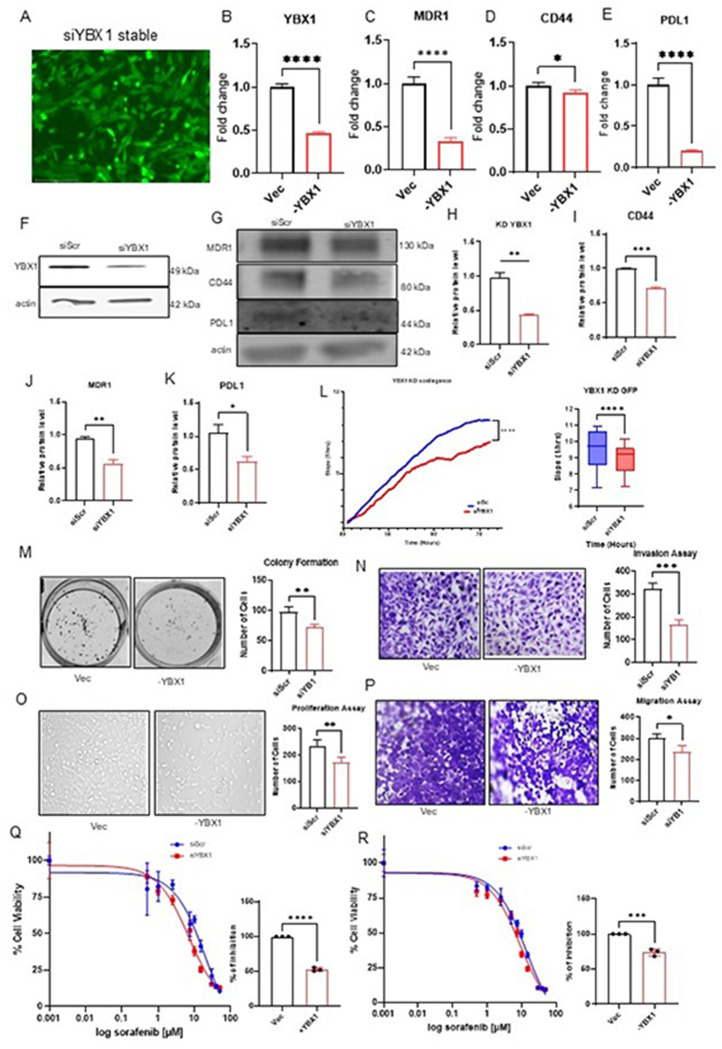
Inhibition of YBX1 attenuates cancer phenotype characteristics and sorafenib sensitivity in HCC A–E) YBX1 knockdown and tumorigenic phenotype of SK-Hep1 cells, with stable knockdown (siYBX1) compared to scrambled control (siScr). (A) Puromycin-selected GFP-enriched cells confirm the stable siYBX1 cell line via FACS. (B–E) mRNA levels of YBX1, PD-L1, CD44, and MDR1 are significantly reduced in YBX1 knockdown cells compared to controls. F–K) Western blot analysis and quantification of YBX1 and related drug resistance markers. (F) Knockdown efficiency of YBX1 confirmed by Western blot. (G) Protein levels of MDR1, CD44, and PD-L1 decrease following YBX1 knockdown. H–K) Quantified protein expression (normalized to β-actin) shows significant reductions in YBX1, CD44, MDR1, and PD-L1 after YBX1 silencing. L) Real-time cell growth curves using the xCELLigence system show decreased proliferation in YBX1 knockdown cells, with a box plot of slope values quantifying proliferation rates. M) Colony formation assay: representative images and quantification reveal a significant decrease in clonogenic potential after YBX1 knockdown. N) Matrigel invasion assay indicates reduced invasiveness of YBX1-silenced cells compared to controls. O) Proliferation assay shows significantly fewer cells in siYBX1-treated samples. P) Migration assay demonstrates impaired cell migration following YBX1 knockdown. Q–R) Sorafenib sensitivity assays post-YBX1 knockdown. (Q) A 24-hour treatment with sorafenib results in a lower IC50 in YBX1-knockdown cells than in scramble controls; the right panel shows decreased fold resistance. (R) Similar results are observed with 48 hours of exposure, confirming YBX1 knockdown reduces sorafenib resistance. Right panels: bar graphs illustrate shifts in IC50. Data are presented as mean ± SE. Groups were compared using an unpaired t-test (*p < 0.05; ** p < 0.01; *** p < 0.001; **** p < 0.0001).

Our results showed a positive correlation between YBX1 overexpression and increased tolerance to sorafenib treatment in HCC. Further, we investigated the impact of YBX1 knockdown on sorafenib sensitivity in HCC. Four different siRNAs were used to select the most efficient siRNA to be used for the YBX1 knockdown (Supplementary Table 1). Stable siYBX1 knockdown lines were further established using puromycin selection and FACS enrichment (Fig. 4A; GFP expressing plasmids Supplementary Figure S2 and S3). To validate the functional significance of YBX1 in HCC progression, we performed molecular and phenotypic characterization of YBX1-knockdown stable cell lines. RT-qPCR analysis confirmed a significant reduction in YBX1 mRNA expression in siYBX1 cells compared to siScr (scramble) controls (Fig. 4B). In parallel, the expression of key drug resistance-associated genes, PD-L1, MDR1, and CD44, was significantly downregulated in siYBX1 knockdown cells (Fig. 4C–E) as compared to the scramble control. Western blot analysis confirmed efficient knockdown of YBX1 protein and reduced expression of PD-L1, MDR1, and CD44 at the protein level (Fig. 4F–K), reinforcing the transcriptional data. As expected, YBX1 knockdown significantly inhibited tumorigenic capacity, including colony formation, proliferation, xCELLigence analysis, migration, and invasion (Fig. 4L–P), further demonstrating YBX1’s role in promoting malignancy in HCC.

We evaluated the IC_50_ values of YBX1 knockdown cell lines treated with increasing concentrations of sorafenib (0.5–50 μM) for 24 and 48 hours, which were significantly lower than those of scramble controls. Specifically, at 24 hours, the siYBX1 cells exhibited an IC_50_ of 11.47 μM, compared with 6.39 μM in scramble controls, representing a 47.27% decrease (Fig. 4Q). At 48 hours, this difference widened, with YBX1 KD cells showing an IC_50_ of 8.5 μM compared to 6.47 μM in scramble controls, representing a 24.6% decrease (Fig. 4R). These results indicate that YBX1 knockdown confers a significant decrease in sorafenib sensitivity at 24 and 48 hrs., implicating YBX1 in sorafenib resistance. Together, these findings demonstrate that YBX1 is a key regulator of HCC proliferation, tumor progression, and metastatic potential, and that it plays a pivotal role in driving sorafenib resistance in HCC.

### YBX1 and drug resistance markers are enriched in the generated sorafenib-resistant HCC cell lines

**Figure 5:**
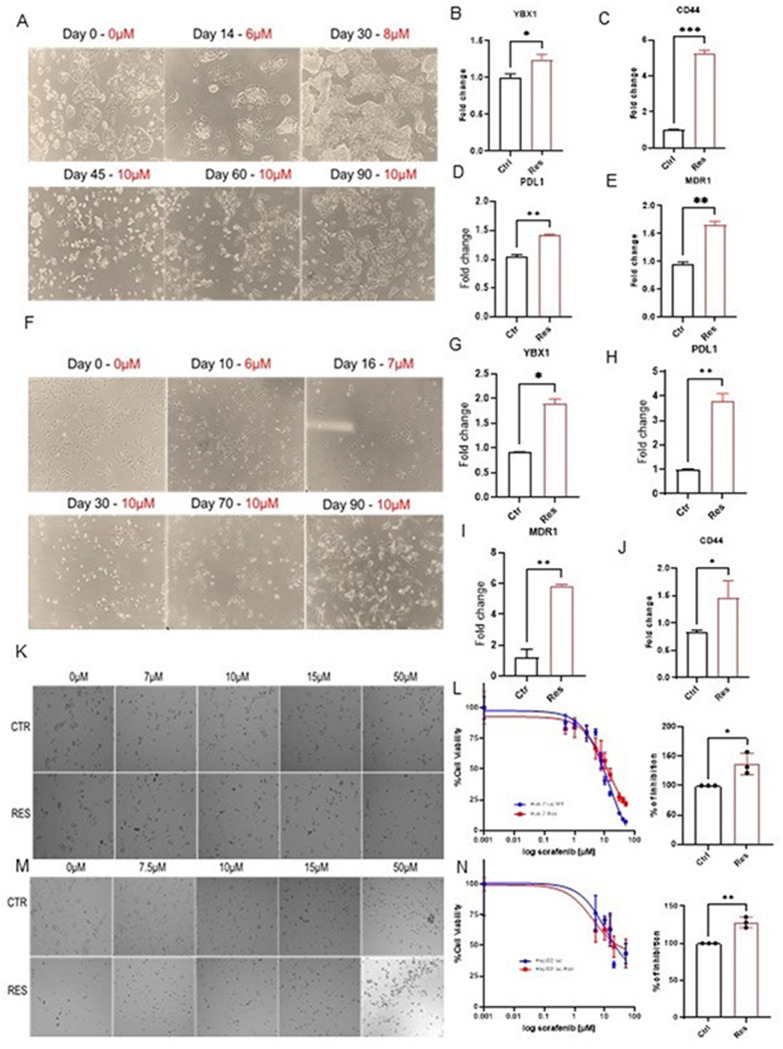
YBX1 and associated drug-resistant markers are upregulated in sorafenib-resistant HCC cell lines compared to parental cell lines A) Phase-contrast images illustrate the stepwise development of a sorafenib-resistant HepG2-luc cell line. Cells were gradually exposed to increasing concentrations of sorafenib (6–10 μM) over 90 days to establish stable resistance. B–E) qRT-PCR analysis compares gene expression levels between parental HepG2-luc cells (Ctrl) and sorafenib-resistant HepG2-luc cells (Res). Expression of YBX1 (B), CD44 (C), PD-L1 (D), and MDR1 (E) is significantly upregulated in resistant cells, indicating acquisition of a drug-resistant phenotype. F) Phase-contrast images show the development of sorafenib-resistant Huh7 cells, gradually adapted to 10 μM sorafenib over 90 days. G–J) RT-qPCR analysis reveals significant upregulation of YBX1 (G), PD-L1 (H), MDR1 (I), and CD44 (J) in resistant Huh7 cells compared to parental controls. K) Representative phase-contrast images of HepG2-luc parental (Ctrl) and resistant (Res) cells treated with increasing concentrations of sorafenib (0–50 μM) for 24 hours. L) Dose–response curves demonstrate that sorafenib-resistant HepG2-luc cells have significantly higher IC50 values, confirming reduced drug sensitivity. The right panel quantifies IC50 shifts. (M) Phase-contrast images of Huh7 parental (Ctrl) and resistant (Res) cells treated with sorafenib (0–50 μM) for 24 hours. N) Sorafenib dose–response curves further confirm significantly elevated resistance in Huh7-resistant cells, as shown by right-shifted IC50 curves. The bar plot compares viability inhibition. Collectively, these data demonstrate that sorafenib resistance in HCC is associated with increased YBX1 expression and induction of multiple drug resistance markers (CD44, MDR1, PD-L1) in both HepG2 and Huh7 models. Data are presented as mean ± SE. The comparison between groups was performed using an unpaired t-test (*p < 0.05; ** p < 0.01; *** p < 0.001; **** p < 0.0001).

To determine whether YBX1 contributes to acquired drug resistance, we developed sorafenib-resistant HCC cell lines by gradually exposing HepG2-luc and Huh7-luc cells to 10 μM sorafenib. The resulting cell lines, HepG2@10 μM and Huh7@10 μM, displayed sustained resistance and were cultured continuously under sorafenib pressure (Fig. 5A and 5F). qRT-PCR analysis revealed a significant increase in YBX1 mRNA levels in both resistant cell lines compared to their respective parental controls (Fig. 5B and 5G).

This upregulation was accompanied by elevated expression of drug resistance genes, including MDR1, PD-L1, and CD44, in HrpG2 luc-resistant (Fig. 5C–E) and huh7 luc-resistant (Fig. 5H–J) cell lines compared with the respective parental controls. These results are consistent with our earlier findings and support the hypothesis that YBX1 contributes to sorafenib-induced stress resistance. The generation of sorafenib-resistant cells and the identification of resistance markers at various concentrations revealed an increasing trend in resistance marker levels (supplementary Figure S4).

To determine if the upregulation of YBX1, CD44, and PD-L1 observed in sorafenib-resistant HCC cell lines translates into functional resistance, we performed MTT assays on HepG2@10 μM and Huh7@10 μM cells alongside their respective non-resistant parental controls. Both cell lines were treated with increasing concentrations of sorafenib (0–50 μM) for 24 hours. Results showed that sorafenib-resistant cells exhibited significantly higher IC_50_ values than their non-treated counterparts, indicating greater tolerance (phase-contrast images at 24 hrs.; Fig. 5K and M). After 24 hours, HepG2@10μM had an IC_50_ value of 27.69μM as compared to 21.63 μM for HepG2 non-treated control (Fig. 5L). Also, Huh7@10μM recorded a 12.06 μM IC_50_ value as compared to 9.17 μM (Fig. 5N). YBX1 has been shown in previous studies to bind the promoters of CD44, PD-L1, and MDR1, thereby upregulating their expression and mediating drug resistance [34, 35]. Our results confirm that continuous sorafenib treatment of HCC cells leads to YBX1 overexpression, thereby upregulating CD44, PD-L1, and MDR1 and mediating sorafenib resistance in HCC. These results strongly support the crucial role of YBX1 in sorafenib resistance in HCC. These IC_50_ shifts, alongside the previously observed overexpression of YBX1, CD44, PD-L1, and MDR1 in these resistant lines (Fig. 5B–E& G–J), confirm that the acquisition of drug resistance is associated with molecular upregulation of YBX1 and its downstream resistance markers, reflecting an adaptive resistance phenotype.

#### SU056 increased sorafenib sensitivity and reduced tumor burden by inhibiting YBX1 in vivo.

**Figure 6:**
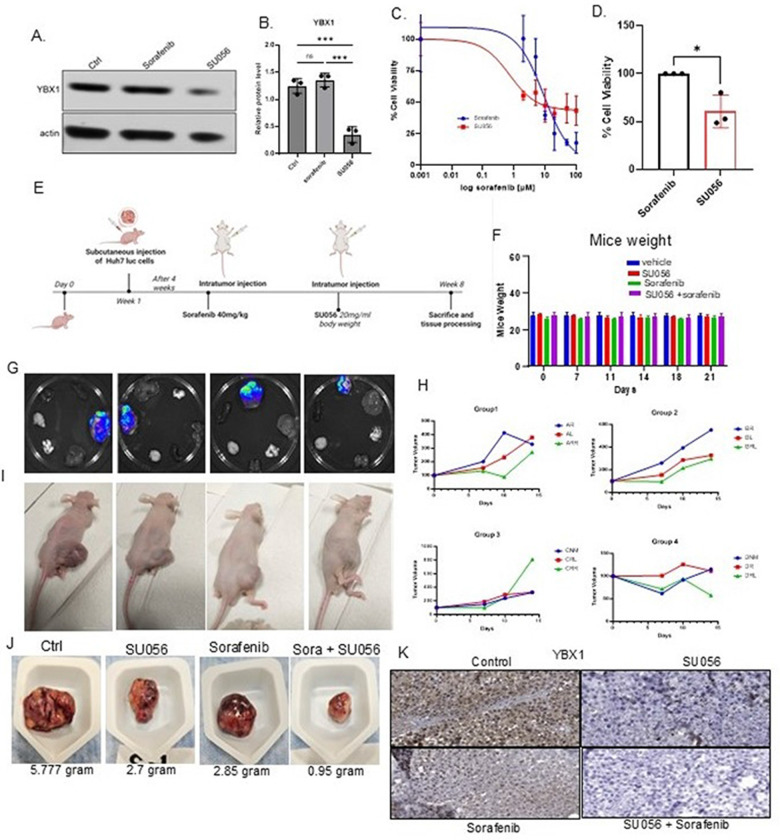
A small molecule, SU056, enhances sorafenib sensitivity by inhibiting YBX1 and reducing tumor burden in HCC A–B) Western blot analysis of YBX1 expression in Huh7 luciferase cells treated with vehicle, sorafenib, and SU056. (B) The representative blot and corresponding quantification show that SU056 significantly reduces YBX1 protein levels, while sorafenib alone does not. (C) A dose-response curve of Huh7 cells treated with sorafenib, with or without SU056, for 24 hours shows increased sensitivity (lower IC50) when SU056 is combined with sorafenib. D) The right panel shows a bar plot comparing cell viability inhibition between treatments. E) Schematic of the in vivo treatment protocol: Mice were subcutaneously injected with Huh7-luc cells, followed by intratumoral administration of sorafenib (40 mg/kg) and/or SU056 (20 mg/ml). Tumors and tissues were harvested in week 8. F) Body weight of the mice was monitored across all treatment groups (vehicle, SU056, sorafenib, and combination); no significant weight loss was observed, indicating tolerability. G) Representative bioluminescence imaging (BLI) of tumor-bearing mice in different treatment groups. The combination treatment resulted in the lowest signal intensity. H) Tumor growth was monitored over time across the four experimental groups, showing a significant reduction in tumor volume in the sorafenib + SU056 group. I) Gross images of mice from each group highlight reduced tumor burden with combination therapy. J) Tumor masses resected from mice show significantly smaller tumors in the combination group (sorafenib + SU056: 0.95 g) compared to controls and monotherapies. K) Immunohistochemistry analysis of tumor sections demonstrates marked suppression of YBX1 protein levels in the combination treatment group versus sorafenib alone, consistent with Western blot results. These data support the idea that SU056 sensitizes HCC tumors to sorafenib by suppressing YBX1, thereby reducing tumor growth and drug resistance. Data are presented as mean ± SE. The comparison between groups was performed using an unpaired t-test (*p < 0.05; ** p < 0.01; *** p < 0.001; **** p < 0.0001).

To further explore the role of YBX1, we used azopodophyllotoxin (SU056), a small molecule that interacts with YBX1 and inhibits its activity [35]. Western blot analysis confirmed that SU056 significantly reduced YBX1 protein levels compared with control and sorafenib-only treatment (Fig. 6A&B), indicating that SU056 can directly antagonize YBX1 expression. To study this effect, we incubated Huh7-Luc cells with sorafenib at different concentrations and 5 μM SU056 for 24 hours to determine sorafenib IC50 values. After 24 hours, the IC50 value for SU056 decreased to 5.57 μM, compared with 11.20 μM for sorafenib alone, resulting in a significant 50% drop (Fig. 6C&D). These results suggest that SU056 lowers the effective concentration required to inhibit cell viability, supporting its role as a sensitizing agent in sorafenib-resistant HCC.

To evaluate the effect of SU056 on sorafenib resistance, a Huh7-luciferase xenograft mouse model was established. After subcutaneous tumor formation, mice were divided into four treatment groups: vehicle control, SU056 alone, sorafenib alone, and sorafenib plus SU056. Intratumoral injections were administered accordingly (Fig. 6E). Body weight changes are minimal across all groups, indicating good tolerability (Fig. 6F). In vivo imaging and tumor volume assessments showed a substantial decrease in tumor size in the sorafenib and SU056 combination group (Fig. 6G–I). Gross tumor analysis revealed the lowest tumor weights in the SU056 + sorafenib group (Fig. 6J), and IHC confirmed reduced YBX1 protein levels post-treatment (Fig. 6K). These results demonstrate that YBX1 inhibition with SU056 sensitizes HCC cells to sorafenib.

## DISCUSSION

The incidence of hepatocellular carcinoma has been linked to chronic hepatitis B and C infections. Improvements in the treatment of hepatitis B and hepatitis C have reduced the incidence of HCC [36]. HCC remains a challenging clinical problem because of its heterogeneity, late diagnosis, and limited response to therapy. Although sorafenib remains the first-line treatment for advanced HCC, resistance typically emerges within six months [37], sharply curtailing its therapeutic benefit [9]. Despite efforts over the past few years to enhance HCC’s sensitivity to sorafenib, overall treatment outcomes remain unsatisfactory [38]. This underscores the urgent need to identify the molecular drivers of resistance and to understand the mechanisms that mediate sorafenib resistance [19].

YBX1 is a well-known multifunctional oncoprotein and has been identified as a prognostic clinical biomarker in various cancers [39]. Our study identifies YBX1 as a critical driver of sorafenib resistance and tumor progression in HCC. By mining TCGA- HCC cohort data (including CPTAC) and independent TMA and cDNA arrays, we observed that YBX1 is consistently overexpressed in HCC at both the mRNA and protein levels. YBX1 expression positively correlates with tumor stage, grade, nodal metastasis, and inflammatory status. These findings implicate YBX1 as a reliable prognostic marker and a driver of malignant progression. Figure 1 shows that YBX1 expression increases from stage I to III, which aligns with increased aggression. Together, these transcriptomic and proteomic findings, along with our histological validation, suggest that YBX1 is not merely a bystander marker but is actively involved in tumor pathogenesis, potentially facilitating the transition from low-grade to aggressive disease. Moreover, the strong correlation between YBX1 expression and features such as nodal metastasis, cirrhosis, and inflammation underscores its role in aggressive tumor phenotypes and potential involvement in the tumor microenvironment [39]. These findings suggest that YBX1 may function not only as a marker of malignancy but also as a driver of hepatocarcinogenesis.

Transient functional studies (Fig. 2) and stable functional studies (Figs. 3 and 4) confirmed that YBX1 overexpression enhances tumor cell viability, proliferation, and sorafenib resistance. These findings align with previous studies linking YBX1 overexpression to aggressive malignant phenotypes, suggesting that YBX1 plays a critical role in driving the invasive potential of HCC cells [40]. Knockdown of YBX1 markedly sensitizes cells to treatment and reduces malignant behaviors, such as migration and colony formation. Notably, YBX1 overexpression increased the sorafenib IC_50_, whereas its silencing restored drug sensitivity. These findings were observed in both transient and stable expression systems, providing robust evidence of YBX1’s oncogenic function. Previous studies have strongly linked elevated YBX1 expression to lower survival rates in patients with head and neck squamous cell carcinoma (HNSCC), underscoring its contribution to tumor cell proliferation, migration, and invasion in both in vitro and in vivo models. [33]. Similarly, in myeloid leukemia, YBX1 upregulation has been linked to increased resistance to apoptosis, reduced differentiation, and impaired leukemic capacity. Deletion of YBX1 alters the expression of apoptosis-related genes, leading to m6A-dependent mRNA decay of MYC and BCL2. Collectively, this evidence suggests that YBX1 functions as a key survival factor across diverse cancer types. In our study, we con^rmed that YBX1 enhances the survival and viability of HCC cell lines in the presence of sorafenib. Sorafenib facilitates apoptosis, mitigates angiogenesis, and suppresses tumor cell proliferation by targeting BRAF, Raf-1, Flt3, VEGFR-2/3, and PDGFR-β [9]. Our experiments demonstrated that YBX1 overexpression significantly increased the IC50 of HCC cells treated with sorafenib, whereas YBX1 knockdown decreased cell viability under similar conditions. These results indicate that YBX1 contributes to sorafenib resistance by enhancing cell survival and proliferation.

Mechanistically, YBX1 upregulated key drug-resistance-associated genes MDR1, PD-L1, and CD44 at both the mRNA and protein levels (Fig. 3), and these genes were downregulated in YBX1 knockdown cell lines (Fig. 4). These markers are well-established contributors to immune evasion, multidrug efflux, and cancer stemness. Knockdown of YBX1 reversed this expression pattern and abrogated tumorigenic traits (Fig. 4), confirming that YBX1 orchestrates a multidimensional resistance program in HCC. Multidrug resistance (MDR) significantly hampers effective cancer treatment, causing many deaths among patients undergoing targeted therapies [41, 42]. The MDR mechanisms include faster xenobiotic metabolism, enhanced drug export, increased production of some growth factors, improved DNA repair, and multiple genetic alterations, including gene mutations, amplifications, and epigenetic alterations [42]. MDR is a major obstacle for targeted therapies in liver cancer due to the high phenotypic and molecular heterogeneity of liver cancer cells [43]. HCC patients have been shown to develop MDR during therapy, which leads to poor survival and prognosis [43]. Additionally, CD44 is a major cell-matrix- associated protein whose expression has been linked to enhanced tumor aggressiveness, metastasis, and resistance to various therapies [44]. CD44 has been shown in previous studies to upregulate P-gp (ABCB1), a transporter that mediates drug resistance [45]. A recent meta-analysis concluded that CD44 expression correlated with Tumor, Node, Metastasis (TNM) stage in HCC and that positive CD44 expression was associated with poorer overall survival than CD44-negative expression [44]. Studies have demonstrated that continuous treatment of HCC cell lines with sorafenib can induce CD44 expression, which is associated with increased sorafenib resistance. [46]. Additionally, programmed cell death ligand-1 (PD-L1) is an immune checkpoint protein that interacts with programmed cell death-1 (PD-1), which is expressed on activated T cells and other immune cells and has been used in cancer therapy, including HCC [47]. PD-L1 expression increases in tumor-infiltrating immune cells (TIIC) in HCC tissues after sorafenib treatment [48]. An increase in PD-L1 expression in tumor-infiltrating immune cells (TIICs) has been linked to HCC progression after sorafenib treatment [48]. When the PD-1/PD-L1 pathway is activated, it suppresses immune cell function and promotes the production of specific cytokines, such as IFN-γ, which, in turn, weakens the T cell response to tumors. [47]. The nuclear localization of YBX1 has been shown to transcriptionally regulate the expression of MDR1, CD44, and PDL1, thereby inducing drug resistance [34, 35, 49]. Proteomic approaches for characterizing the YBX1 protein in conjunction with long non-coding RNAs reveal target interactions that contribute to drug resistance [50].

To examine whether YBX1 plays a role in acquired resistance, we established sorafenib-resistant cell lines (Fig. 5). These resistant cells showed elevated YBX1 expression, along with increased expression of MDR1, CD44, and PD-L1. The parallel upregulation of these markers in resistant lines and in YBX1-overexpressing models strongly supports a causal relationship between YBX1 expression and acquired drug resistance [51]. The enrichment of YBX1 and its downstream resistance markers in sorafenib-adapted HCC cells highlights its adaptive function under therapeutic pressure. As resistance emerged, YBX1 expression increased, triggering the transcriptional upregulation of MDR1, PD-L1, and CD44, key effectors of chemoresistance and immune evasion. These data parallel our stable overexpression and knockdown models, supporting the causal involvement of YBX1 in acquired resistance. These observations underscore YBX1’s utility as both a predictive biomarker and a functional target for overcoming therapeutic resistance in HCC. Additionally, our data align with broader patterns observed in solid and hematologic malignancies, where YBX1 promotes evasion of apoptosis, stemness, and treatment failure. For instance, in leukemia, YBX1 deletion disrupts the expression of survival genes such as MYC and BCL2 by promoting m6A-mediated mRNA decay. These cross-cancer insights reinforce the notion of YBX1 as a universal mediator of resistance [52].

The in vivo results presented here highlight the therapeutic potential of SU056 in modulating sorafenib sensitivity by inhibiting YBX1. In xenograft models, SU056 alone moderately reduced tumor growth, but in combination with sorafenib, it markedly reduced tumor burden and viability. This suggests that inhibiting YBX1 synergizes with sorafenib to overcome YBX1-mediated resistance. Immunohistochemical and molecular analyses revealed decreased YBX1 expression in SU056-treated tumors, supporting the hypothesis that YBX1 is a central mediator of tumor progression and therapeutic resistance. Notably, combination therapy achieved superior tumor suppression without significant toxicity, as evidenced by stable body weight in mice. These ^ndings underscore the critical role of YBX1 in hepatocellular carcinoma and highlight the utility of SU056, a YBX1 inhibitor, as an adjuvant to existing targeted therapies such as sorafenib.

Collectively, our findings demonstrate that YBX1 is a key mediator of sorafenib resistance in HCC, promoting the expression of resistance-associated genes, including CD44, PD-L1, and MDR1. YBX1 overexpression not only drives tumor aggressiveness but also significantly elevates sorafenib IC_50_ values, underscoring its role in drug tolerance. Conversely, targeted inhibition or knockdown of YBX1 sensitizes HCC cells to sorafenib, both in vitro and in vivo. Treatment with the small-molecule YBX1 inhibitor SU056 effectively downregulated YBX1 expression and lowered IC_50_ values in sorafenib-resistant HCC models, thereby reducing tumor burden and improving drug efficacy. These results support the concept that targeting the YBX1 axis, particularly in tumors co-expressing CD44, PD-L1, and MDR1, may represent a promising therapeutic strategy to overcome resistance. By integrating transcriptomic, proteomic, and functional data, our study positions YBX1 as both a predictive biomarker and a druggable vulnerability in HCC. Therapeutic strategies targeting YBX1 may resensitize resistant tumors to sorafenib, curb aggressive tumor behaviors, and ultimately improve survival outcomes in patients with advanced HCC.

## CONCLUSION

This study highlights YBX1 as a central mediator of drug resistance and tumor progression in HCC, a malignancy with rising global incidence and limited therapeutic success. Through integrative transcriptomic, proteomic, and functional analyses, we demonstrate that YBX1 is significantly overexpressed in HCC tissues at both the mRNA and protein levels. Its expression positively correlates with tumor stage, grade, metastatic potential, and poorer patient outcomes, establishing YBX1 as a potential biomarker of disease aggressiveness.

Functionally, YBX1 confers resistance to sorafenib, the first-line systemic therapy for advanced HCC. YBX1 overexpression increased IC_50_ values and enhanced oncogenic properties, including proliferation, migration, and colony formation, whereas YBX1 knockdown sensitized cells to sorafenib and significantly suppressed these malignant traits. Moreover, sorafenib-resistant cell lines exhibited increased YBX1 levels and coordinated upregulation of CD44, PD-L1, and MDR1, all of which are established contributors to immune evasion, metastasis, and multidrug resistance.

Our results further reveal that pharmacological inhibition of YBX1 with SU056 markedly sensitized HCC cells to sorafenib, both in vitro and in vivo. The combination treatment effectively reduced tumor burden and YBX1 expression without observable toxicity, suggesting a promising therapeutic strategy. These findings underscore YBX1’s importance not only as a biological marker but also as a therapeutic target in HCC, with potential to resensitize sorafenib-resistant cells. Importantly, the YBX1-CD44/PD-L1/MDR1 axis is a critical signaling node underlying sorafenib resistance. By modulating this network, YBX1 orchestrates a survival program that undermines drug efficacy. Inhibiting YBX1 is a compelling strategy to overcome treatment resistance and improve patient outcomes.

Looking ahead, these findings call for further mechanistic studies to elucidate the transcriptional and post-transcriptional roles of YBX1 in therapeutic resistance. Targeted disruption of YBX1-driven signaling may represent a viable approach to re-sensitize resistant HCC tumors to sorafenib and potentially other therapies. As such, YBX1 stands out as a clinically relevant target with translational potential to reshape treatment paradigms for patients with advanced hepatocellular carcinoma.

## Supplementary Material

This is a list of supplementary files associated with this preprint. Click to download.

• SupplemnentaryFiles.docx

## Figures and Tables

**Table 1:** Demographic and pathological characteristics for Liver Cancer-Tissue micro-Array (LV8011a/b).

Variable	Value
Total, n	81
*Demographics*	
Age, Median (IQR)	50 (21–98)
Sex, n (%)	
Male	37.1% (30)
Female	62.9% (51)
*Pathologic Characteristics*	
Primary Tumor (pT), n (%)	
T1	1 (1.5)
T2	17 (21)
T3	23 (29)
T4	1 (1.5)
TX (None Analyzed)	38 (47)
AJCC Prognostic Stage Group, n (%)	
I	1 (1.5)
II	17 (21.2)
IIA	0 (0)
IIB	0 (0)
IIIA	22 (27.2)
IIIB	0 (0)
IIIC	1 (1.5)
IV	0 (0)
IVA	1 (1.5)
IVB	0 (0)
Organ/Anatomic Site	
Liver	75 (92)
Skin	1 (1.5)
Abdominal Cavity	1 (1.5)
Pelvic Cavity	1 (1.5)
Chest Wall	2 (2)
Colon	1 (1.5)

**Table 2:** Demographic and pathologic characteristics for LiverCancer-CDNA Array I (LVRT101).

Variable	Value
Total, n	48
*Demographics*	
Age, Median (IQR)	66 (21–86)
Sex, n (%)	
Male	72.9% (35)
Female	27.1% (13)
*Pathologic Characteristics*	
Primary Tumor (pT), n (%)	
T1	13 (27.1)
T2	12 (25)
T3	17 (35.4)
T4	0 (0)
TX (None Analyzed)	6 (12.5)
Regional Lymph Nodal Status (pN), n (%)	
N0	8 (16.7)
N1	0 (0)
N2	0 (0)
NX (None Analyzed)	40 (83.3)
Distant Metastases (pM), n (%)	
M0	0 (0)
M1	3 (6.2)
MX (None Analyzed)	45 (93.8)
AJCC Prognostic Stage Group, n (%)	
I	13 (27.1)
II	12 (25)
IIA	0 (0)
IIB	0 (0)
IIIA	16 (33.3)
IIIB	0 (0)
IIIC	0 (0)
IV	3 (6.3)
IVA	0 (0)
IVB	0 (0)
Undefined	4 (8.3)
Histological Grade, n (%)	
Well Differentiated	10 (20.8)
Moderately Differentiated	21 (43.8)
Poorly Differentiated	7 (14.6)
Undifferentiated	10 (20.8)

## Abbreviations

BBB: Blood–brain barrier penetration
CSD: Cold shock domain
CPTAC: Clinical Proteomic Tumor Analysis Consortium
EMT: Epithelial–mesenchymal transition
FACS: Fluorescence-activated cell sorting
GFP: Green Fluorescence Protein
HCC: Hepatocellular carcinoma
MDR1: Multi-drug resistant 1
MTT: 3-(4,5-dimethylthiazol-2-yl)-2,5-diphenyltetrazolium bromide)
SEER: Epidemiology and End Results
TKIs: Tyrosine-kinase inhibitors
TMA: Tissue microarray
VEGFA: Vascular endothelial growth factor A
YBX1: Y box binding protein
